# NiSe_2_/Ni(OH)_2_ Heterojunction Composite through Epitaxial-like Strategy as High-Rate Battery-Type Electrode Material

**DOI:** 10.1007/s40820-020-0392-8

**Published:** 2020-02-21

**Authors:** Hao Mei, Zhaodi Huang, Ben Xu, Zhenyu Xiao, Yingjie Mei, Haobing Zhang, Shiyu Zhang, Dacheng Li, Wenpei Kang, Dao Feng Sun

**Affiliations:** 1 College of Science, China University of Petroleum (East China), Qingdao, 266580 Shandong People’s Republic of China; 2 School of Material Science and Engineering, China University of Petroleum (East China), Qingdao, 266580 Shandong People’s Republic of China; 3 Key Laboratory of Structural Chemistry, Fujian Institute of Research on the Structure of Matter, Chinese Academy of Sciences, Fuzhou, 350002 People’s Republic of China; 4 Key Laboratory of Eco-chemical Engineering, Ministry of Education Laboratory of Inorganic Synthesis and Applied Chemistry, College of Chemistry and Molecular Engineering, Qingdao University of Science and Technology, Qingdao, 266402 Shandong People’s Republic of China; 5 Shandong Provincial Key Laboratory of Chemical Energy Storage and Novel Cell Technology, School of Chemistry and Chemical Engineering, Liaocheng University, Liaocheng, 252059 Shandong People’s Republic of China

**Keywords:** Supercapacitor, Heterojunction, Epitaxial-like growth, NiSe_2_/Ni(OH)_2_

## Abstract

Constructing heterojunction is a promising way to improve the charge transfer efficiency and can thus promote the electrochemical properties. Herein, a facile and effective epitaxial-like growth strategy is applied to NiSe_2_ nano-octahedra to fabricate the NiSe_2_-(100)/Ni(OH)_2_-(110) heterojunction. The heterojunction composite and Ni(OH)_2_ (performing high electrochemical activity) is ideal high-rate battery-type supercapacitor electrode. The NiSe_2_/Ni(OH)_2_ electrode exhibits a high specific capacity of 909 C g^−1^ at 1 A g^−1^ and 597 C g^−1^ at 20 A g^−1^. The assembled asymmetric supercapacitor composed of the NiSe_2_/Ni(OH)_2_ cathode and p-phenylenediamine-functional reduced graphene oxide anode achieves an ultrahigh specific capacity of 303 C g^−1^ at 1 A g^−1^ and a superior energy density of 76.1 Wh kg^−1^ at 906 W kg^−1^, as well as an outstanding cycling stability of 82% retention for 8000 cycles at 10 A g^−1^. To the best of our knowledge, this is the first example of NiSe_2_/Ni(OH)_2_ heterojunction exhibiting such remarkable supercapacitor performance. This work not only provides a promising candidate for next-generation energy storage device but also offers a possible universal strategy to fabricate metal selenides/metal hydroxides heterojunctions.

**Electronic supplementary material:**

The online version of this article (10.1007/s40820-020-0392-8) contains supplementary material, which is available to authorized users.

## Highlights

- A facile and effective epitaxial-like growth strategy is applied to fabricate the NiSe_2_/Ni(OH)_2_ heterojunction composite.
- The assembled asymmetric supercapacitor based on the heterojunction composite surpasses most of the reported results. It is the first time that the powdered electrode materials can have such large capacity, high rate, and extreme long cycle life.

**Electronic supplementary material:**

The online version of this article (10.1007/s40820-020-0392-8) contains supplementary material, which is available to authorized users.

## Graphical abstract

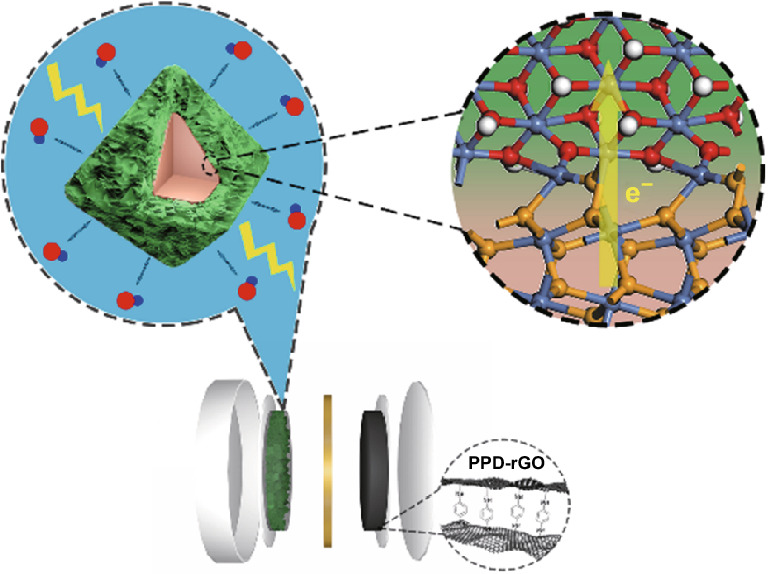

## Introduction

Due to the severe consumption of fossil energy and environmental issues, it is urgent to develop novel clean-energy technologies, including solar, wind, and tide [1]. After harvesting, these energy usually cannot directly parallel to the grids due to their severe fluctuations, thus energy storage devices affording large charging/discharging currents and long cycle lives are urgently needed [2–5]. Scientists have paid much efforts to develop energy storage devices, such as lithium/sodium/potassium ion batteries, fuel cells, and electrochemical supercapacitors [6–10]. Among them, supercapacitors are attractive due to their high safety, long cycle lives, large power densities, and low cost [11–13]. However, their low energy densities, compared with other electrochemical energy technologies, limit their further applications [14–16]. Several methods have been utilized to improve the energy storage performance of supercapacitor, and fabricating asymmetric supercapacitor is believed to be an ideal solution [17–19]. An asymmetric supercapacitor is composed of an electric double-layer capacitive (EDLC) electrode, enabling the fast energy delivery, and a pseudo-capacitive/battery-type electrode, ensuring large energy storage [20–22]. Therefore, an asymmetric supercapacitor can inherit both the advantages of large power densities from EDLC and large energy densities from pseudo-capacitive/battery-type electrode [23].

Ideal electrode material for high-performance supercapacitor should have the following features: (1) high capacity; (2) excellent rate capability; (3) long cycle lives [24]. Ni(OH)_2_ is one of the most attractive materials due to its large theoretical capacity, easy-synthesis, and environmental friendly [25–27]. However, it still suffers from the low conductivity, which is harmful to the rate performance [28]. Furthermore, the large volume expansion during charging/discharging process leads to cycling issues [29–31]. One promising strategy to improve its supercapacitor performance is compositing Ni(OH)_2_ with other materials. Generally, the Ni(OH)_2_-based composite materials should have the following features. First, a considerable electron conductivity is required to compensate the low conductivity of Ni(OH)_2_ for improving the rate capability [31]. Second, a high mechanical stability is necessary for the cycling performance [32]. Moreover, the electron transfer between different phases should be fast and efficient [33]. However, it is difficult to satisfy all the features. For instance, the most widely reported Ni(OH)_2_/carbon composite materials have good conductivity and mechanical strength [34–36]. But the van de Waals interaction between Ni(OH)_2_ and carbon largely limits the electron transfer efficiency between them. Recently, the composite of Ni(OH)_2_ and Ni oxides/sulfides/phosphides was investigated as promising electrode materials, and the heterojunction structures between Ni(OH)_2_ and Ni oxides/sulfides/phosphides are an important reason contributing to their remarkable supercapacitance behaviors [37–39]. However, there are still two issues needed to be addressed. Ni oxides/sulfides/phosphides do not perform good conductivity, much lower than carbon-based materials. Furthermore, oxidation/sulfofication/phosphorization treatments on porous Ni(OH)_2_ are the commonly used methods, and the cycling issue is not addressed due to the relatively fragile porous Ni(OH)_2_ basis [37]. Therefore, it is urgent to find a proper material and a proper synthesis route to obtain desired Ni(OH)_2_-based composite materials for supercapacitor applications.

NiSe_2_ is one semiconductor with the low resistivity below 10^−3^ Ω cm^−1^ [39]. Using proper synthesis routes, NiSe_2_ single-crystal nano-octahedra can be easily obtained and are expected to perform good conductivity with high mechanical strength due to its single-crystal feature. If porous Ni(OH)_2_ can be properly composited to the NiSe_2_ nano-octahedra, an ideal electrode material can be obtained, although there is no research reporting such composite as far as we know. Herein, we report an novel epitaxial-like growth strategy on the fabrication of NiSe_2_/Ni(OH)_2_ composite materials with NiSe_2_ nano-octahedra as the precursor. Through the treatment process, Ni atoms at the surfaces of NiSe_2_ nano-octahedra are released to the solution and coordinated to OH^−^ ions. And those OH^−^ ions simultaneously bond to the unreleased surface Ni atoms, then a close NiSe_2_/Ni(OH)_2_ heterojunction can be formed in an epitaxial-like crystallization route. Under proper reaction conditions, the obtained NiSe_2_/Ni(OH)_2_ electrode material exhibits the following advantages: (1) the heterojunction can improve electron transfer by DFT calculations; (2) large specific surface areas and suitable microporous structure ensure the abundant electrochemical active sites which are easily accessed by the electrolyte and rapid ion migration within the electrode; (3) the highly crystallized NiSe_2_ nano-octahedra foundations provide high mechanical strength, thus the good cycling stability was obtained. As a result, the NiSe_2_/Ni(OH)_2_ electrode material obtained under optimized conditions displays the outstanding electrochemical performances of high specific capacity of 909 C g^−1^ and good cycling stability of 85% capacity retention after 5000 cycles. The asymmetric supercapacitor composed of NiSe_2_/Ni(OH)_2_ cathode and p-phenylenediamine-functional reduced graphene oxide (PPD-rGO) anode exhibits ultrahigh specific capacity of 303 C g^−1^ and remarkable energy density of 76.1 Wh kg^−1^ at the power density of 906 W kg^−1^, as well as the excellent cycling stability of 82% capacity retention after 8000 cycles, demonstrating it a promising supercapacitor device.

## Experiment Section

### Materials Preparation

#### Preparation of NiSe_2_ Precursor

All the chemical regents are directly used without any further purification. In a typical procedure, 0.5 mmol nickel nitrate and 3 mmol selenium powder were dissolved in 10 mL deionized water and 10 mL hydrazine hydrate solution, respectively, and mixed together. Then, the mixed solution was stirred for 30 min to obtain the clear solution. The solution was sealed in a 50 mL Teflon-lined stainless container and maintained 140 °C for 24 h. After that, the precipitation was collected by filtration, washed by deionized water and ethanol for several times, and dried at 60 °C for 24 h. Finally, the NiSe_2_ precursor was obtained.

#### Preparation of NiSe_2_/Ni(OH)_2_

In our experiment, 0.1 g NiSe_2_ precursor was dispersed into 20 mL 0.1 M KOH to obtain the homogeneous solution. Then, 1 mL 30% H_2_O_2_ was added into the solution. The mixture solution was sealed in a 50 mL Teflon-lined stainless container and maintained 170 °C for different times. The reaction product was collected by filtration, washed by deionized water and ethanol for several times, and dried at 60 °C for 24 h. For convenience, for products at different reaction times, the NiSe_2_/Ni(OH)_2_ composite was termed as NiSe_2_/Ni(OH)_2_-1h, NiSe_2_/Ni(OH)_2_-2h, NiSe_2_/Ni(OH)_2_-3h, and NiSe_2_/Ni(OH)_2_-6h, respectively. For comparison, 80 mg NiSe_2_ precursor and 20 mg Ni(OH)_2_ (Acros, for analysis) were physically mixed to obtain the NiSe_2_/Ni(OH)_2_ and termed as NiSe_2_/Ni(OH)_2_-Grind.

#### Preparation of PPD-rGO

The preparation of PPD-rGO is based on the previous reported works [40]. 50 mg GO was dispersed in 50 mL deionized water to form a homogeneous solution. Then, 54 mg p-phenylenediamine was added into the solution and stirred for 30 min. The mixed solution was sealed into autoclave at 180 °C for 12 h. The obtained products were washed by deionized water for several times.

### Materials Characterization

Powder X-ray diffraction (XRD) was used to investigate the phase purity and crystallinity of prepared samples (Cu Kα = 0.15418 nm). X-ray photoelectron spectroscopy (XPS) was employed to examine the surface chemical states. The morphology and microstructure were examined by scanning electron microscopy (SEM) and transmission electron microscopy (TEM). Thermogravimetric analysis (TGA) tests were performed on a Mettler Toledo TGA instrument under O_2_ condition at a heating rate of 10 °C min^−1^. The N_2_ adsorption/desorption curves and pore size distributions were collected from surface area analyzer ASAP-2020.

### DFT Calculations

DFT calculations were performed using CASTEP in Material Studio software package [41–43]. The Ni(OH)_2_ (110) plane was cleaved and placed on the NiSe_2_ (100) plane. Six layers of Ni(OH)_2_ (110) planes and three layers of NiSe_2_ (100) planes were used, and a vacuum slab of 10 Å was added at each side to build the NiSe_2_(100)/Ni(OH)_2_(110) heterojunction structure. The exchange–correlation functional of GGA + PBESOL is utilized for optimizing the constructed model and DOS calculations. The cutoff energy of 780 eV was used, and the norm conserving pseudopotentials were used for each type of atom. Due to the existence of Ni atoms, spin polarization was considered. FFT grid of 48 × 48 × 48 and SCF tolerance of 1 × 10^−5^ eV/cell was used. This set is adequate for our calculations.

### Electrochemical Measurements

All the electrochemical performances were tested by CHI 760e instrument. In our experiment, we used a Ni foam as current collector, platinum gage as counter electrode, 6 M KOH as electrolyte solution. For working electrode, 16 mg activated materials, 2 mg conductive carbon black, and 40 μL 5% polytetrafluoroethylene (mass radio, 8:1:1) were mixed together to get a homogenous slurry. Then, 2.5 mg of the mixture was painted on the Ni foam. Electrochemical impedance spectroscopy (EIS) was tested by using a disturbance voltage in a frequency range of 0.01–10^6^ Hz. In our work, the button asymmetric supercapacitor was assembled through using 2.5 mg NiSe_2_/Ni(OH)_2_-2h of as the anode, 4 mg PPD-rGO as the cathode.

## Results and Discussion

### Mechanisms of Epitaxial-like Growth of NiSe_2_/Ni(OH)_2_ on NiSe_2_ Nano-octahedra

**Fig. 1:**
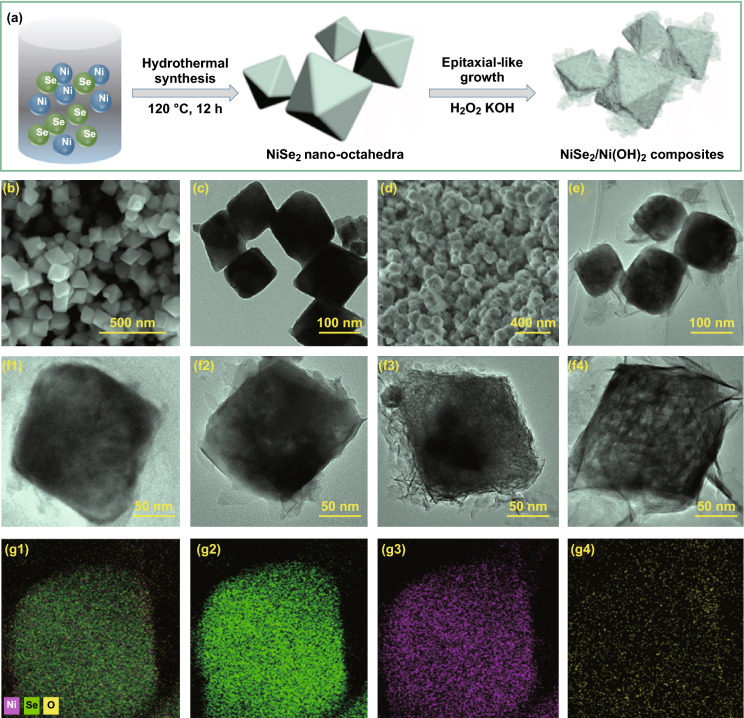
**a** Synthetic process of NiSe_2_/Ni(OH)_2_ heterojunction composites. **b**, **c** SEM and TEM images of NiSe_2_. **d**, **e** SEM and TEM images of NiSe_2_/Ni(OH)_2_-2h. **f**(1–4) TEM images of NiSe_2_/Ni(OH)_2_-1h, NiSe_2_/Ni(OH)_2_-2h, NiSe_2_/Ni(OH)_2_-3h, NiSe_2_/Ni(OH)_2_-6h. **g**(1–4) EDS mapping images of NiSe_2_/Ni(OH)_2_-2h

The overall synthetic process of NiSe_2_/Ni(OH)_2_ heterojunction composite is shown in Fig. 1a. Initially, using nickel nitrate and selenium powder, NiSe_2_ nano-octahedra were successfully prepared through a simple and controllable hydrothermal synthesis route. Subsequently, under the oxidative and alkaline conditions, NiSe_2_ nano-octahedra were converted to NiSe_2_/Ni(OH)_2_ composite. As shown in Fig. 1b, c, NiSe_2_ precursor exhibits a distinct octahedral feature in a size of 100–200 nm, and no other impurities were found. Figure 1c shows the TEM image of NiSe_2_ octahedra, which well-matches the SEM image. The surface morphology and structure of NiSe_2_ nano-octahedra have distinctly changed after the oxidation–hydrolysis treatment as displayed in Figs. 1d, e and S1. It is clear that the outer of NiSe_2_ nano-octahedra is modified and surrounded by thin nanoflakes. The selected electron diffraction patterns were further employed to verify the constituent (Fig. S1). The inner and outer of nano-octahedra display the bright spots and rings feature, respectively, which illustrate that epitaxial-like growth of polycrystalline Ni(OH)_2_ nanoflakes on the surfaces of monocrystalline NiSe_2_ nano-octahedra.

For revealing the transformation of the NiSe_2_/Ni(OH)_2_ composites, we investigate the morphology changes of NiSe_2_/Ni(OH)_2_ composites at different reaction time, and the results are shown in Fig. 1f_1_–f_4_. When the reaction time reaches 1 h, a small amount of nanoflakes appear on the surfaces of nano-octahedra (Fig. 1f_1_). At 2 h, the corners of nano-octahedra are corroded and a large amount of ultrathin Ni(OH)_2_ nanoflakes crystallize and spread on the surfaces (Fig. 1f_2_). However, NiSe_2_/Ni(OH)_2_-2h still presents the octahedral shape. The EDS mapping shown in Fig. 1g_1_–g_4_ demonstrates that the inner octahedra of NiSe_2_/Ni(OH)_2_-2h are still NiSe_2_, consistent with the selected electron diffraction patterns, while the oxygen is well spread at the outer. When the reaction time reaches 3 h, it is clear that the octahedral block has been severely corroded and a quite number of thick nanosheets are formed (Fig. 1f_3_). After 6 h of reaction time, NiSe_2_ nano-octahedra have been almost completely destroyed, and a large amount of thick nanosheets constructed into the octahedral shape (Fig. 1f_4_).

**Fig. 2:**
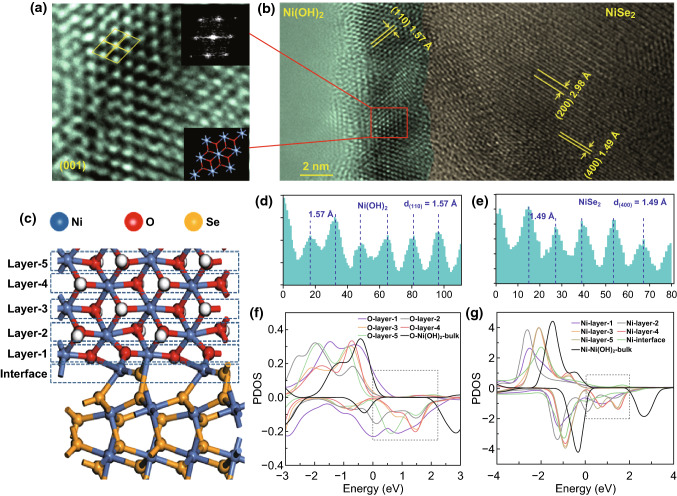
**a**, **b** HRTEM images of NiSe_2_/Ni(OH)_2_ heterojunction. **c** The constructed NiSe_2_/Ni(OH)_2_ model for calculations. **d**, **e** Profile plots of the calibration for measuring the spacing in panels. **f**, **g** PDOS of O and Ni atoms at different layers

The epitaxial-like growth of the Ni(OH)_2_ nanoflakes at the surfaces of the NiSe_2_ nano-octahedras contributes to the heterojunction structure of NiSe_2_/Ni(OH)_2_, as demonstrated in the HRTEM images (Fig. 2). The Ni(OH)_2_ and NiSe_2_ domains can be clearly observed. Figure 2a is the zoom-in Ni(OH)_2_ domain, and the hexagonal spots in the FFT image (the inset at the up-right corner) indicate that the Ni(OH)_2_ domain is top-view of the (001) plane (the inset as the bottom-right corner). The zoom-in NiSe_2_ domain shown in Fig. S2 is the top-view of the (001) plane due to the square spots, although slight distortion can be noticed. The measured lattice fringes at Ni(OH)_2_ and NiSe_2_ domains are ascribed to the Ni(OH)_2_ (110) planes and the NiSe_2_ (400) planes, and the distances are 1.57 and 1.49 Å, respectively (Fig. 2d, e). The epitaxial-like crystallization of Ni(OH)_2_ on the NiSe_2_ requires the similar lattice constant between them. As shown in Fig. S1, the (110) plane of Ni(OH)_2_ is composed of the Ni–O octahedral layer with the distance between two Ni atoms equaling to 5.39 Å, nearly twice of the distance between two Ni atoms on the NiSe_2_ (001) plane, it is likely that the epitaxial-like growth of Ni(OH)_2_ on NiSe_2_ contributing to the NiSe_2_-(100)/Ni(OH)_2_-(110) heterojunction.

The heterojunction can effectively facilitate the electron transportation at the interfaces. DFT calculation was therefore performed to investigate the density of the electron state of the atoms at the NiSe_2_-(100)/Ni(OH)_2_-(110) interface. The model build for the calculation is shown in Fig. 2c, and the partial density of states (PDOS) for each atom was calculated, as shown in Figs. 2f, g and S3. At the interface, the Ni atoms present typical conductive feature due to no forbidden gap in their PDOSs, and furthermore, the PDOSs of Ni and O atoms in Ni(OH)_2_ layers all present no forbidden gap, indicating the good electron conductivity at the interface, as well as a few layers of Ni(OH)_2_ out of the interface. However, it is obvious that the forbidden gap tends to open in the PDOSs of Ni and O when Ni(OH)_2_ layers are away from the interface. In layer 5, Ni and O atoms exhibit similar PDOSs compared with Ni(OH)_2_. The PDOSs of Ni and Se atoms within the NiSe_2_ layers close to the interface were also calculated, and they all present conductive feature with no forbidden gaps appear; as shown in Fig. S3, indicating the formation of NiSe_2_/Ni(OH)_2_ heterojunction does not influence the conductivity of NiSe_2_. The DFT calculations well demonstrate the superiority of the NiSe_2_/Ni(OH)_2_ heterojunction for electron transport.

**Fig. 3:**
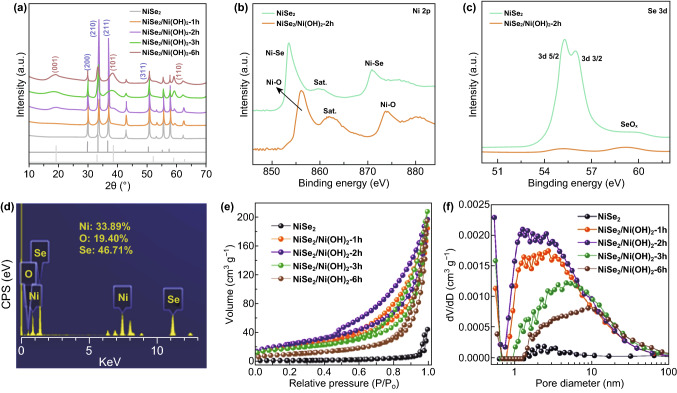
**a** XRD pattern of NiSe_2_ and NiSe_2_/Ni(OH)_2_ composite with different reaction times. **b** Ni 2p of NiSe_2_ and NiSe_2_/Ni(OH)_2_-2h. **c** Se 2p of NiSe_2_ and NiSe_2_/Ni(OH)_2_-2h. **d** EDX spectrum of NiSe_2_/Ni(OH)_2_-2h. **e** N_2_ adsorption/desorption curves. **f** Pore size distribution curves

The XRD patterns of NiSe_2_ and NiSe_2_/Ni(OH)_2_ heterojunction composites at different reaction time are presented in Fig. 3a. It is clear that all prepared samples exhibit strong NiSe_2_ peaks, and Ni(OH)_2_ peaks gradually increase with reaction time. The characteristic diffraction peaks at 29.80°, 33.41°, 36.70°, and 50.48° represent the (200), (210), (211), and (311) planes of NiSe_2_, respectively. After treated in hydrogen peroxide and potassium hydroxide aqueous solution, the characteristic diffraction peaks of Ni(OH)_2_ appear. Interestingly, Ni(OH)_2_ peaks arose firstly at 2*θ* value of 59.05°, associated with the (110) planes of Ni(OH)_2_. While the diffraction peaks at 19.26° and 38.54° belong to (001) and (101) planes, which are stronger than other peaks in the PDF standard card and for most reported Ni(OH)_2_ nanomaterials, appear later than the (110) peak [44]. We suggest that the abnormally prior-growth of (110) peak is associated with the epitaxial-like crystallization of Ni(OH)_2_ on the (110) plane.

XPS spectra were further collected to get insight into the surface chemical state changes during the treatments, as shown in Figs. 3b, c and S5. For the NiSe_2_ precursor, the characteristic peaks of 853.52 and 870.94 eV are indexed to Ni 2p_3/2_ and Ni 2p_1/2_ due to the Ni–Se bond, and there is only negligible Ni–O peaks due to the trace NiO_*x*_ on the NiSe_2_ surfaces [45]. After immersing into the KOH/H_2_O_2_ aqueous solution for 2 h, a noticeable change in the surface chemical state can be observed as demonstrated in the NiSe_2_/Ni(OH)_2_-2h XPS spectrum. The characteristic peaks at 856.04 and 873.93 eV are assigned to Ni 2p_3/2_ and Ni 2p_1/2_ due to Ni–O bond, indicting the formation of external Ni(OH)_2_. Meanwhile, the Se 3d peak almost disappear in the NiSe_2_/Ni(OH)_2_-2h XPS spectrum while is strong in the NiSe_2_ spectrum (Fig. 3c), implying the outer NiSe_2_ has converted to Ni(OH)_2_. It is worth mentioning that inner part of the NiSe_2_ octahedra maintains since the XRD pattern of NiSe_2_/Ni(OH)_2_-2h still presents strong NiSe_2_ characteristic peaks.

Based on the XRD and XPS results, we suggest the two steps of epitaxial process from NiSe_2_ to NiSe_2_/Ni(OH)_2_ composites: initially, Se^−^ ion is oxidized by hydrogen peroxide, and Ni^2+^ ions are released into solutions. Subsequently, unreleased Ni atoms at the surfaces are coordinated to OH^−^ ions forming into a thin layer Ni(OH)_2_ with (110) planes due to the restriction of NiSe_2_ (100) planes. Meanwhile, the Ni^2+^ and OH^−^ ions in solution also precipitate on the Ni(OH)_2_ (110) plane, contributing to the epitaxial-like route and the growth of (110) peak in the XRD pattern. Eventually, the NiSe_2_/Ni(OH)_2_ composites with NiSe_2_-(100)/Ni(OH)_2_-(110) heterojunction are achieved.

For better understanding the conversion process from NiSe_2_ nano-octahedra to NiSe_2_/Ni(OH)_2_ heterojunction composites, we obtained the products from either pure hydrogen peroxide aqueous solution or pure potassium hydroxide aqueous solution. As illustrated in Fig. S4a, NiSe_2_ in neither pure H_2_O_2_ solution nor pure KOH solution can be transformed to the desired NiSe_2_/Ni(OH)_2_ compositions. In the absence of H_2_O_2_, NiSe_2_ retained the original phase without any change. Without KOH, the product is in multi-phase, and no Ni(OH)_2_ peaks can be observed. Furthermore, we also immersed NiO into the KOH/H_2_O_2_ aqueous solution to demonstrate that the Ni(OH)_2_ is originated from NiSe_2_ rather than the trace NiO on the NiSe_2_ surfaces. As shown in Fig. S4b, NiO maintains unchanged during the treatments. Therefore, we can conclude that immersing NiSe_2_ in the KOH/H_2_O_2_ aqueous solution results in the epitaxial-like growth of NiSe_2_/Ni(OH)_2_ heterojunction composites.

Quantitatively analysis in the amounts of NiSe_2_ and Ni(OH)_2_ within the NiSe_2_/Ni(OH)_2_ is based on the EDX and TGA measurements. The EDX spectrum of NiSe_2_/Ni(OH)_2_-2h is displayed in Fig. 3d. The atom ratio of Ni, O, Se is 33.89%, 19.40%, 46.71%, respectively, and the molar ratio between NiSe_2_ and Ni(OH)_2_ is 2.4:1. However, due to that the EDX only focuses on a small spot, and it is irrational to conclude that the molar ratio within the whole sample is the same. Therefore, TGA tests were further employed to quantitatively investigate the compositional features of all NiSe_2_/Ni(OH)_2_ composites. As shown in Fig. S6 and Table S1, it is clearly that the mass fraction of Ni(OH)_2_ increased with the reaction time, and the molar ratio between NiSe_2_ and Ni(OH)_2_ within NiSe_2_/Ni(OH)_2_-2h is 1.7:1, which is close than the value from EDX. At 6 h, most of the NiSe_2_ has transformed to Ni(OH)_2_, and the molar ratio between NiSe_2_ and Ni(OH)_2_ is 0.387:1, associated with the TEM results shown in Fig. 1f4. The TGA results clearly demonstrate that the growth of Ni(OH)_2_ on NiSe_2_ can be easy regulated depending on the treatment time.

Figure 3e displays the N_2_ adsorption/desorption curves of the prepared samples. Obviously, NiSe_2_ nano-octahedra show an extremely low N_2_ adsorption capacity even at 1.0 *P*/*P*_0_, implying its low specific surface associated with the well crystallized phase. However, after a controllable epitaxial-like growth, the obtained NiSe_2_/Ni(OH)_2_ composites exhibit much higher N_2_ adsorption, indicating their enlarged specific surface areas. The calculated specific surface areas based on the N_2_ adsorption/desorption isotherms of NiSe_2_, NiSe_2_/Ni(OH)_2_-1h, NiSe_2_/Ni(OH)_2_-2h, NiSe_2_/Ni(OH)_2_-3h and NiSe_2_/Ni(OH)_2_-6h are 4.29, 73.59, 88.17, 63.59, and 36.29 m^2^ g^−1^, respectively. Interestingly, NiSe_2_/Ni(OH)_2_-2h performs the largest surface areas, implying an non-proportional relation between reaction time and the porosity. The corresponding pore size distribution of prepared samples is displayed in Fig. 3f. Illustrated by the curves of NiSe_2_, NiSe_2_/Ni(OH)_2_-1h, and NiSe_2_/Ni(OH)_2_-2h, the pore volume increases regularly in a range of 1–3 nm at the initial 2 h. When the reaction time is above 3 h, the pore volume in the size from 1 to 5 nm drastically reduces. Considering in that the molar ratio of NiSe_2_ and Ni(OH)_2_ is 1.41:1 for 3 h and 0.39:1 for 6 h, and it is highly likely that the further crystallization of Ni(OH)_2_ after 2 h blocks the smaller pores. Therefore, we suggest an optimized reaction time is necessary to achieve larger surface areas and proper pore structures for supercapacitor applications, and here, NiSe_2_/Ni(OH)_2_-2h is the most promising one compared with its counterparts.

### Electrochemical Analysis

**Fig. 4:**
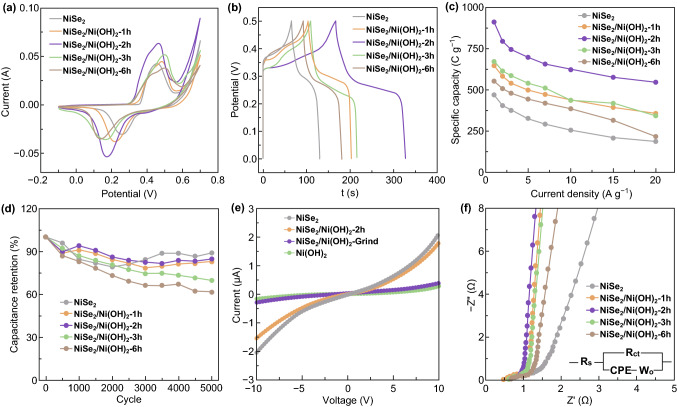
**a** CV curves of prepared electrode at 5 mV s^−1^. **b** GCD curves of prepared electrode at 5 A g^−1^. **c** Comparison of specific capacity at different current density. **d** Cycling performance at 5 A g^−1^. **e** I–V characterizations of prepared samples. **f** Nyquist plot of prepared electrode

The unique structure of NiSe_2_/Ni(OH)_2_ composites implies its potential as supercapacitor electrode material. Thus, for investigating the supercapacitance performances of NiSe_2_/Ni(OH)_2_ composites, a series of electrochemical characterizations including cyclic voltammetry (CV), galvanostatic discharge–charge (GCD), and electrochemical impedance spectroscopy (EIS) were employed, and the results are shown in Figs. 4 and S7. The CV curves of NiSe_2_ and all NiSe_2_/Ni(OH)_2_ composites are presented at a scan rate of 5 mV s^−1^. We can observe a pair of strong redox peaks, especially, NiSe_2_/Ni(OH)_2_-2h exhibits the strongest redox peaks, and the good symmetry of its redox peaks indicates the high Coulomb efficiency. The related electrochemical reactions were as follow (Eqs. 1 and 2) [46]:

1 $${\text{NiSe}}_{2} + {\text{OH}}^{ - } \leftrightarrow {\text{NiSe}}_{2} {\text{OH}} + {\text{e}}^{ - }$$

2 $${\text{Ni}}\left( {\text{OH}} \right)_{2} + {\text{OH}}^{ - } \leftrightarrow {\text{NiOOH}} + {\text{H}}_{2} {\text{O}} + {\text{e}}^{ - }$$

The specific capacities of all NiSe_2_/Ni(OH)_2_ electrode materials were calculated based on the GCD curves, and the results are presented in Fig. 4c. Corresponding to the CV curves, all GCD curves presented symmetric potential platforms resulted from reversible redox reactions (Fig. 4b). As expected, NiSe_2_/Ni(OH)_2_-2h shows much longer charge–discharge time, demonstrating its larger capacity than its counterparts. The specific capacity of NiSe_2_/Ni(OH)_2_-2h reaches a high value of 909 C g^−1^ (1818 F g^−1^) at the current density of 1 A g^−1^. Furthermore, at the current density of 20 A g^−1^, the specific capacity of NiSe_2_/Ni(OH)_2_-2h is 597 C g^−1^ (1194 F g^−1^), illustrating its good rate capability at large charge–discharge currents. Figure 4d shows the cycling tests of NiSe_2_ and all NiSe_2_/Ni(OH)_2_ composites at the current density of 5 A/g. NiSe_2_, NiSe_2_/Ni(OH)_2_-1h, NiSe_2_/Ni(OH)_2_-2h, NiSe_2_/Ni(OH)_2_-3h, and NiSe_2_/Ni(OH)_2_-6h present 89%, 84%, 85%, 69%, and 61% present capacity retention after 5000 cycles, respectively. Interestingly, NiSe_2_, NiSe_2_/Ni(OH)_2_-1h, and NiSe_2_/Ni(OH)_2_-2h all present higher stability than other NiSe_2_/Ni(OH)_2_ composites synthesized with longer reaction time. Based on the TEM results in Fig. 1, we suggest longer reaction time that destroys the NiSe_2_ octahedra foundation, leading to the unstable structures. Thus, it is concluded that the excellent stability is attributed to the high mechanical strength of NiSe_2_ nano-octahedra. Furthermore, the electrochemical performance of NiSe_2_/Ni(OH)_2_-2h is competitive with other reported materials which is shown in Table S4, indicating tremendous potential as high-performance electrode materials.

I–V characteristic curves were examined, and the results are shown in Fig. 4e. It is apparent that NiSe_2_ nano-octahedra and NiSe_2_/Ni(OH)_2_-2h exhibit preferable electron conductivity, much better than Ni(OH)_2_. Especially, it is noteworthy that the NiSe_2_/Ni(OH)_2_-grind, which is prepared by simply mixing and grinding NiSe_2_ and Ni(OH)_2_ (the weight ratio of Ni(OH)_2_ is 20%, same to the value in NiSe_2_/Ni(OH)_2_-2h) does not perform good conductivity. Therefore, it is concluded that the NiSe_2_/Ni(OH)_2_ heterojunction structure facilitate the electron transport, and the conductivity of NiSe_2_ is well inherited in the NiSe_2_/Ni(OH)_2_ heterojunction composite.

Electrochemical impedance spectra were collected in a frequency range of 0.01–10^6^ Hz and displayed in Fig. 4f. Obviously, the impedance spectra of all electrode materials perform similar curves, which composed of a semicircle in the high-frequency region and a sloping straight line in the low-frequency region. The impedance spectra of all electrodes were fitted using the equivalent circuit presented in the inset of Fig. 4f. *R*_s_ and *R*_ct_ are the electrolyte resistance and faradic resistance. On account of the depressed semicircle regions, CPE (constant phase angle element) was chosen rather than capacitor. Wo (Warburg element) was adopted to investigate the diffusion of electrolyte within the electrode. As listed in Table S2, the CPE-T values represent the EDLCs, and the CPE-P closing to 1 suggests the small leakage of current. It is apparent that NiSe_2_/Ni(OH)_2_-2h exhibit lower Rct of 0.222 Ω and Wo-R of 0.601 Ω compared with its counterparts, illustrating the rapid electrolyte diffusion and fast faradic reaction.

For better understanding the charge–discharge behavior of electrode materials, the scan rates and oxide peak current were fitted using Eq. 3 [21]:

3 $$i = av^{b}$$

The fitting results are presented in Fig. S8 and Table S3. Obviously, *b*-values of all the electrode materials are close to 0.5, indicating the diffusion-controlled behavior and battery-type charge–discharge process. It is worth mentioning that Eq. 3 is originated from Eq. 4 [21]:

4 $$i = i_{\text{EDLC }} + i_{\text{diff}} = k_{1} v + k_{2} v^{0.5}$$

In this formula, *k*_1_*v* and *k*_2_*v*^0.5^ are the capacitive contribution (*i*_EDLC_) and diffusion contribution (*i*_diff_) of current. The capacitive process including physical adsorption/desorption of electrolyte ions and fast surface redox reactions (capacitive contributions), and the diffusion process is kinetic sluggish redox reactions controlled by the diffusion of electrolyte ion (diffusion-controlled contributions). This method is suitable for batteries due to the narrow range of sweep rates and is widely used in many articles [47–49]. For supercapacitors, however, on account of the large range of sweep rate, this method is not appropriate. As shown in Fig. S9, the calculated areas originated from capacitive contribution (*k*_1_*v*) exhibit strange shapes for all electrodes, of which the CVs are collected using the sweep rate from 5 to 25 mV/s. This can be explained by the theory mentioned in Ref. [46]. *i*_EDLC_ and *i*_diff_ can be expressed in details as Eqs. 5 and 6 [50]:

5 $$i_{\text{diff}} = k_{1} v^{0.5} = nFA_{\text{diff}} C_{0} \left( {D_{0} } \right)^{0.5} \left( t \right) A_{\text{diff}} D_{0}^{0.5} , = nFv/RT$$

6 $$i_{{{\text{EDLC}} }} = k_{2} v = A_{\text{EDLC}} C_{d} v A_{\text{EDLC}}$$

Herein, *n* and *F* are the electron number during the reaction (equal to 1 in our case) and faradic constant. *A*_diff_, *C*_0_, and *D*_0_ are the electrochemical active surface area, reactant concentration, and diffusion efficiency of reactant, respectively. *χ* is a dimensionless number. *A*_EDLC_ and *C*_*d*_ are the electrochemical active area of EDLC and the specific capacitance of the double layer with the unit of F cm^−2^, respectively. It is worth mentioning that *χ* is dependent to sweep rate *v*. Therefore, when the sweep rate range is narrow (the peak current does not drastically shift), it is reasonable to treat *χ* as a constant, and thus *k*_2_ is also constant. However, in a wide sweep rate range (the peak current clearly shift with the sweep rate), treating *χ* as constant is not appropriate, and the equation of *i *= *k*_1_*v *+ *k*_2_*v*^0.5^ can only be applied to the peak current rather than the whole CVs [51]. Therefore, *k*_1_*v* and *k*_2_*v*^0.5^ are capacitive and diffusion contribution to the peak current, respectively.

Due to the aforementioned reasons, we used the equation of *i *= *k*_1_*v *+ *k*_2_*v*^0.5^ to fit the peak currents, and the fitting results are presented in Table 1 and Fig. S10. As shown in Table 1, the value of *k*_1_*v* is much lower than *k*_2_*v*^0.5^ in all electrode materials, demonstrating a distinct battery-type behavior. The capacitive contributions (*k*_1_*v*) for all electrodes are similar, while the diffusion contributions (*k*_2_*v*^0.5^) are quite different. NiSe_2_/Ni(OH)_2_-2h performs a distinctly higher *k*_2_*v*^0.5^ value of 0.15, larger than its counterparts, associated with its largest specific capacity, implying the largest amounts of electroactive sites. Considering in that the electroactive sites are mainly from Ni(OH)_2_ and that NiSe_2_/Ni(OH)_2_-2h does not have the largest Ni(OH)_2_ weight ratio, it is reasonable to conclude that the optimized Ni(OH)_2_ amounts are necessary to guarantee the full utilization of the fast electron transportation in the heterojunction. Smaller amounts of Ni(OH)_2_ cannot provide enough active sites. Overgrowth of Ni(OH)_2_ leads to poor porosity and difficult transportation of electrons from active sites to the heterojunction. Both of them are detrimental to the specific capacity and rate performances.

**Table 1:** Fitted results of oxide peak current density versus scan rate (*v* = 0.025 V s^−1^)

	*k*_1_	*k*_2_	EDLC contributions *k*_1_ × *v*	Diffusion contributions *k*_2_ × *v*^0.5^	Reduced Chi-square
NiSe_2_	− 0.083	0.60	− 0.00208	0.0949	4.2 E^−7^
NiSe_2_/Ni(OH)_2_-1h	0.35	0.64	0.00875	0.103	3.7 E^−6^
NiSe_2_/Ni(OH)_2_-2h	0.27	0.95	0.00675	0.150	3.7 E^−6^
NiSe_2_/Ni(OH)_2_-3h	0.21	0.74	0.00525	0.117	2.2 E^−6^
NiSe_2_/Ni(OH)_2_-6h	0.25	0.50	0.00625	0.0791	1.8 E^−6^

We believe the enhancement of the battery-type NiSe_2_/Ni(OH)_2_ heterojunction electrode performances is mainly associated with the synergistic effects of this unique composite material. As is known, the fast ion migration and rapid electronic conductivity are essential for the performance of an electrode. The pure NiSe_2_ nano-octahedra deliver a high electronic conductivity, but the low porosity cannot provide enough electrochemical active sites. After careful treatments, hierarchical porous Ni(OH)_2_ shell forms on the outer surfaces of NiSe_2_, providing abundant electrochemical active sites. Furthermore, the hierarchically porous structure provides rich pores for the ion migration (supported by the BET analysis), and the NiSe_2_/Ni(OH)_2_ heterojunction enabling the fast electron transportation at the interfaces (supported by the DFT calculations). Therefore, due to the formation of the NiSe_2_/Ni(OH)_2_ heterojunction, both the electron conductivity of the NiSe_2_ and the fast ion migration in Ni(OH)_2_ are fully utilized, enabling the remarkable performances of the NiSe_2_/Ni(OH)_2_ heterojunction electrode.

The contribution of NiSe_2_ and Ni(OH)_2_ is also analyzed, and the results are shown in Table S5. It is clearly seen that with the formation of Ni(OH)_2_, the NiSe_2_ contribution to the SC value decreases. We suggest it is due to the decreasing amounts of NiSe_2_. The Ni(OH)_2_ contribution reaches the maximum in NiSe_2_/Ni(OH)_2_-2h, although the Ni(OH)_2_ fraction is larger in NiSe_2_/Ni(OH)_2_-3h and NiSe_2_/Ni(OH)_2_-6h associated with that the over crystallized Ni(OH)_2_ does not present ideal pore structure, confirmed by the BET analyses. The NiSe_2_/Ni(OH)_2_-2h presents the largest specific surface area of 88.17 m^2^ g^−1^.

Therefore, we can conclude that the outstanding performance of NiSe_2_/Ni(OH)_2_-2h is attributed to the following reasons: First, the heterojunction between Ni(OH)_2_ with high electrochemical activity and NiSe_2_ with high conductivity improves the charge transportation within the electrode, enabling higher electrochemical activity. Meanwhile, the large specific surface area and abundant microscopes are preferable for the diffusion and transportation of electrolyte. Furthermore, the NiSe_2_ octahedra foundation with high crystallinity is highly stable, enabling the long charge–discharge life.

### Performances of the NiSe_2_/Ni(OH)_2_-2h//PPD-rGO Asymmetric Supercapacitor

**Fig. 5:**
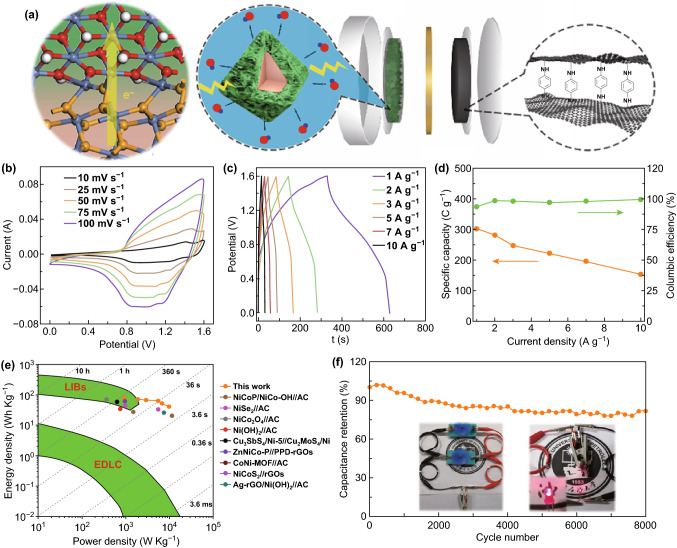
**a** The assembled asymmetric supercapacitor, **b** CV curves, **c** GCD curves, **d** specific capacity and columbic efficiency at different current density, **e** Ragone plot, and **f** cycling performance

The remarkable supercapacitance performance of NiSe_2_/Ni(OH)_2_-2h is due to the porous Ni(OH)_2_ enabling the fast ion migration, the conductive and stable NiSe_2_ octahedra ensuring the fast electron migration and cycling stability, as well as the NiSe_2_/Ni(OH)_2_ heterojunction offering an easy electron transportation from electroactive Ni(OH)_2_ to conductive NiSe_2_. For further investigating, the application of NiSe_2_/Ni(OH)_2_-2h for supercapacitors, a button asymmetric supercapacitor, was fabricated using 2.5 mg NiSe_2_/Ni(OH)_2_-2h as positive electrode and 4 mg PPD-rGO as negative electrode, as illustrated in Fig. 5a. The electrochemical performances of PPD-rGO are illustrated in Fig. S7f, l, and the FT-IR spectrum and the SEM image of PPD-rGO are presented in Fig. S11. The CV curves of PPD-rGO exhibit a typical double-layer capacitance behavior. Even at a high scan rate of 200 mV s^−1^, it still remains a rectangle shape, indicating a fast charge transfer kinetics. The GCD curves of PPD-rGO from 1 to 50 A g^−1^ present a triangular shape and excellent symmetry, suggesting the highly reversible of charge–discharge process. Based on the GCDs, the specific capacity of PPD-rGO is calculated as 504 C g^−1^ (504 F g^−1^), and a high value of 319 C g^−1^ (319 F g^−1^) at 50 A g^−1^ indicates its excellent rate capability.

The electrochemical performances of NiSe_2_/Ni(OH)_2_-2h//PPD-rGO asymmetric supercapacitor are presented in Figs. 5b–f and S12. Calculated from the GCD curves of NiSe_2_/Ni(OH)_2_-2h//PPD-rGO, at a current density of 1 A g^−1^, an ultrahigh specific capacity of 302 C g^−1^ (189 F g^−1^) can be obtained. Furthermore, almost 100% coulomb efficiencies were acquired at different current densities (Fig. 5d). The energy density and power density can be acquired using Eqs. 7 and 8, and the results are shown in Fig. 5e [51]:

7 $$E = \frac{I}{m}\mathop \smallint \limits_{t1}^{t2} V{\text{d}}t$$

8 $$P = \frac{E}{\Delta t}$$

At the power density of 906.4 W kg^−1^, the NiSe_2_/Ni(OH)_2_-2h//PPD-rGO is able to achieve the ultrahigh energy density of 76.1 Wh kg^−1^. Moreover, the NiSe_2_/Ni(OH)_2_-2h//PPD-rGO exhibits a higher energy density and power density than many advanced asymmetric supercapacitors reported recently, such as NiCoP/NiCo-OH//AC [32], NiSe_2_//AC [39], NiCo_2_O_4_//AC [52], Ni(OH)_2_//AC [53], Cu_3_SbS_4_/Ni-5//Cu_2_MoS_4_/Ni [54], ZnNiCo-P//PPD-rGOs [27], CoNi-MOF//AC [55], NiCoS_2_//AC [56], and Ag-rGO/Ni(OH)_2_//AC [57] (Fig. 5e and Table S6). Take the advantage of high energy density and power density, these asymmetric supercapacitors can drive two electric fans for rotation. A red LED (1.6–3 V, 20 mA) 800 can also be lighted using the NiSe_2_/Ni(OH)_2_-2h//PPD-rGO asymmetric supercapacitor device. More importantly, the cycling stability test indicates this asymmetric device can have 82% retention of its original capacity after 8000 cycles. These tests strongly demonstrate the potential practical application of this asymmetric supercapacitor device.

## Conclusions

In summary, using NiSe_2_ nano-octahedra as the precursor, NiSe_2_/Ni(OH)_2_ heterojunction composites with large specific surface areas and rich micropores, as well as good electron conductivity, were successfully constructed through a epitaxial-like growth strategy. The porous Ni(OH)_2_ enables the fast ion migration and large amount of electrochemical active sites, and the NiSe_2_ nano-octahedra offer electron conductivity and mechanical strength for cycling stability. Noteworthy, the NiSe_2_/Ni(OH)_2_ heterojunction providing easy electron transportation from Ni(OH)_2_ to NiSe_2_, confirmed by the DFT calculations, is the domain reason contributing to the synergistic effects. Therefore, the NiSe_2_/Ni(OH)_2_ heterojunction composites obtained under optimized reaction conditions deliver a high specific capacity of 909 C g^−1^ at 1 A g^−1^, an excellent cycling performance of 85% capacity retention after 5000 cycles. Furthermore, the fabricated NiSe_2_/Ni(OH)_2_//PPD-rGO button asymmetric supercapacitors achieve ultrahigh energy density of 76.1 Wh kg^−1^ at 906 W kg^−1^ and outstanding cycling stability of 82% capacity retention after 8000 cycles, indicating tremendous potential in practical application. Our work here provides a novel strategy to synthesize high-performance selenide/hydroxide composites. It needs more efforts to investigate if this method can be applied to other transition metal chalcogenide.

## Electronic Supplementary Material

Below is the link to the electronic supplementary material.

Supplementary material 1 (PDF 1348 kb)
