# Supplementary material for: NiSe2/Ni(OH)2 Heterojunction Composite through Epitaxial-like Strategy as High-Rate Battery-Type Electrode Material
Source: Nanomicro Lett. 2020 Feb 21;12:61. doi: 10.1007/s40820-020-0392-8 (PMC7770911; doi:10.1007/s40820-020-0392-8)
Supplement: Supplementary file 1 — Supplementary material 1 (PDF 1348 kb) [file 40820_2020_392_MOESM1_ESM.pdf]

Supporting Information for

$NiSe_2/Ni(OH)_2$ Heterojunction Composite through Epitaxial-like Strategy as High-rate Battery-type Electrode Material

Hao Mei[1, †], Ben Xu[1, 2, 3, †, *], Zhaodi Huang[1], Zhenyu Xiao[4], Yingjie Mei[2], Haobing Zhang[1], Shiyu Zhang[1], Dacheng Li[5], Wenpei Kang[1, 2], Dao feng Sun[1, 2, *]

[1]College of Science, China University of Petroleum (East China), Qingdao, Shandong 266580, People's Republic of China

[2]School of Material Science and Engineering, China University of Petroleum (East China), Qingdao, Shandong 266580, People's Republic of China

[3]Key Laboratory of Structural Chemistry, Fujian Institute of Research on the Structure of Matter, Chinese Academy of Sciences, Fuzhou 350002, People's Republic of China

[4]Key Laboratory of Eco-chemical Engineering, Ministry of Education Laboratory of Inorganic Synthesis and Applied Chemistry, College of Chemistry and Molecular Engineering, Qingdao University of Science and Technology, Qingdao, Shandong 266402, People's Republic of China

[5]Shandong Provincial Key Laboratory of Chemical Energy Storage and Novel Cell Technology, School of Chemistry and Chemical Engineering, Liaocheng University, Liaocheng, Shandong 252059, People's Republic of China

[†]Hao Mei and Ben Xu contributed equally to this work

*Corresponding authors. E-mail: benxu@upc.edu.cn (Ben Xu), dfsun@upc.edu.cn (Dao feng Sun)

Supplementary Figures

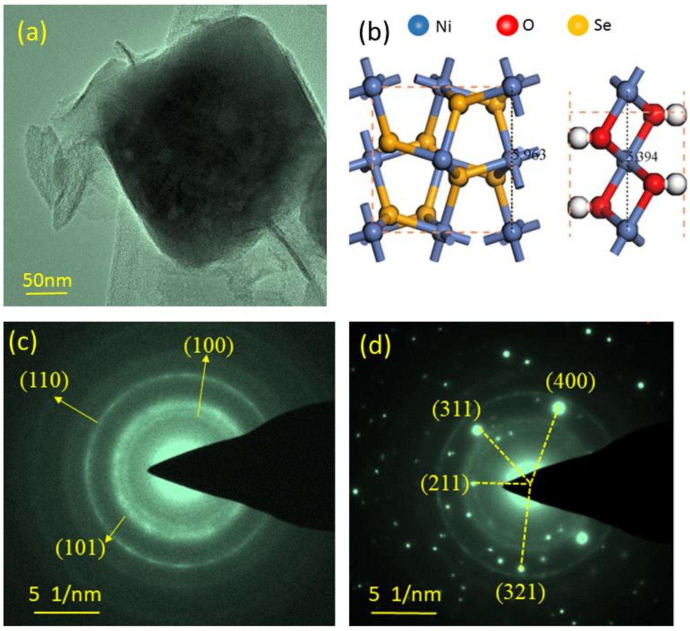

Fig. S1 (**a**) The TEM image of $NiSe_2/Ni(OH)_2$-2h. The $Ni(OH)_2$ nanoflakes grow around the $Ni(OH)_2$ octahedra; (**b**) the crystal structure of $NiSe_2$ (100) and $Ni(OH)_2$ (110). On the $NiSe_2$ (100) plane, the distance between two nearest Ni atoms are 2.98 Å, half of the distance on $Ni(OH)_2$ (110). Therefore, it is likely that the $Ni(OH)_2$ (110) plane can grow on the $NiSe_2$ (100) plane; (**c, d**) The SAED image on $Ni(OH)_2$ nanoflake and $NiSe_2$ octahedra. The SAED rings of the $Ni(OH)_2$ nanoflake indicate its polycrystalline feature. However, the SAED image on the $NiSe_2$ octahedra exhibit clear diffraction spots, indicating the single crystal feature of the $NiSe_2$, and meanwhile, the polycrystalline rings of $Ni(OH)_2$ are also observed, implying the epitaxial growth of $Ni(OH)_2$ on $NiSe_2$.

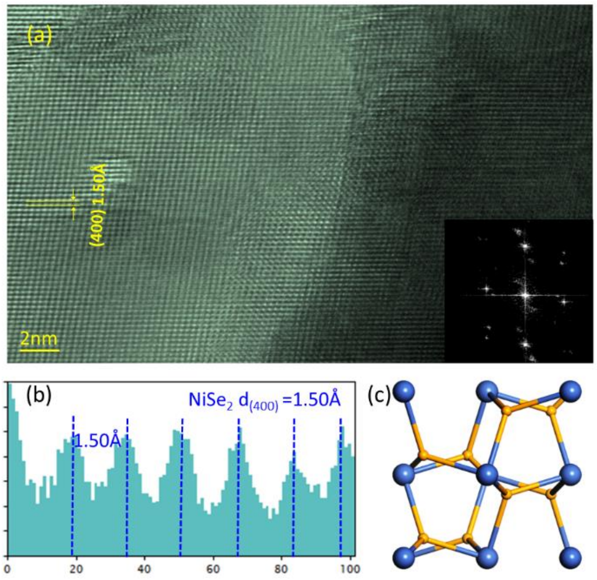

Fig. S2 (**a**) HRTEM images of $NiSe_2$ domain. The inset is the FFT image of the

zoom-in $NiSe_2$ domain, and the square spots imply that this image is the top view of (001) plane of $NiSe_2$; (**b**) Profile plots of the calibration for measuring the spacings in panels; (**c**) The crystal structure of $NiSe_2$ on (001) plane. Ni atoms are in the similar position compared with the spots in FFT image.

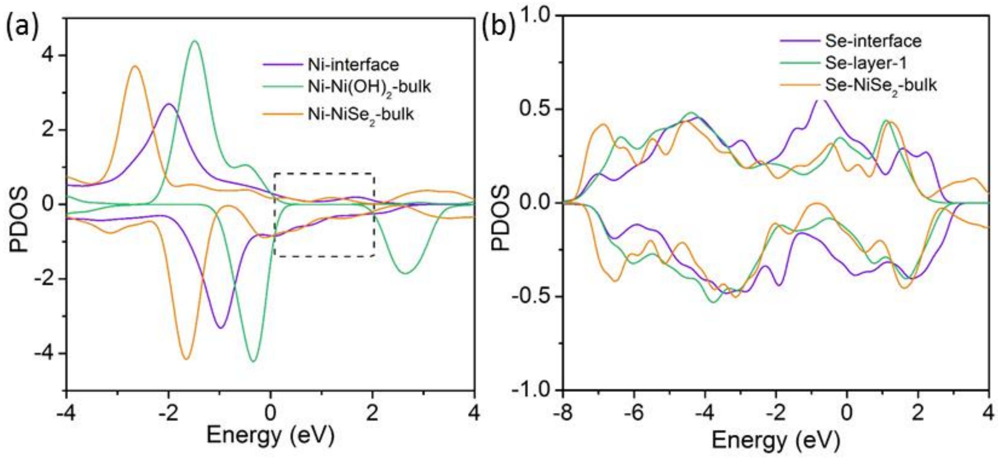

Fig. S3 (**a**) The PDOS of Ni atoms at the $Ni(OH)_2/NiSe_2$ interface and in bulk $NiSe_2$, as well as in bulk $Ni(OH)_2$. It is clear that the PDOS of Ni-$Ni(OH)_2$-bulk presents typical forbidden gap, implying its unsatisfied conductivity. The Ni-interface and Ni-$NiSe_2$-bulk, however, present conductive feature. (**b**) The PDOS of Se atoms at and near the interface, as well as in the bulk. There is no forbidden gap, indicating the conductive feature.

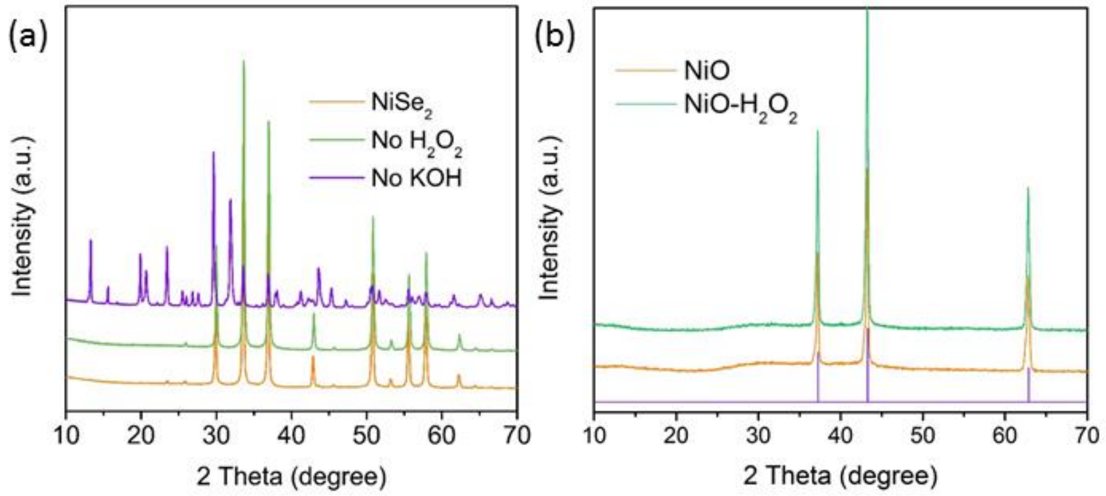

Fig. S4 (**a**) XRD patterns of prepared samples from either pure hydrogen peroxide aqueous solution or pure potassium hydroxide aqueous solution. It is obvious that $NiSe_2/Ni(OH)_2$ composite cannot be produced under either condition. (**b**) XRD patterns of untreated and treated NiO in the H_2O_2 and KOH solution. It is obvious that NiO maintains unchanged.

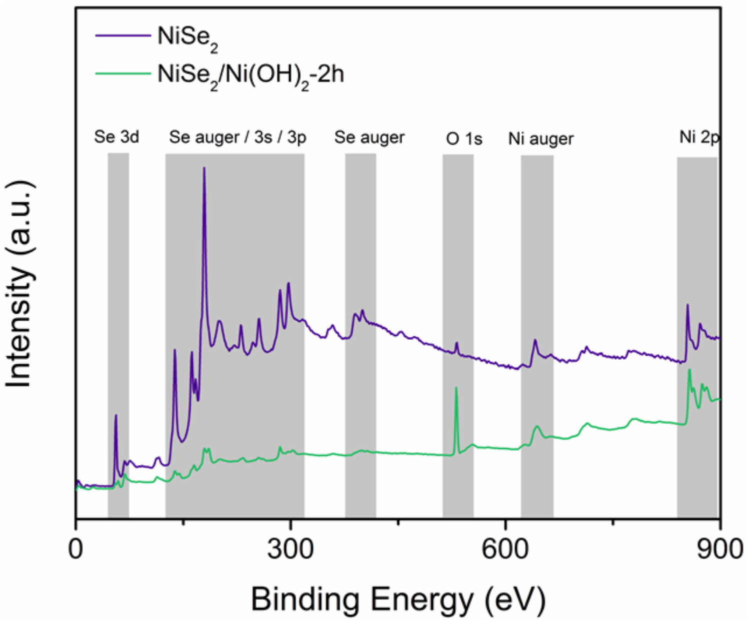

Fig. S5 XPS spectra of $NiSe_2$ and $NiSe_2/Ni(OH)_2$-2h. We can clearly observe a series of characteristic peaks from 0 to 450 eV, which are assigned to Se 3s, Se 3p, Se 3d, and Se auger. The characteristic peak at 531.85 eV of $NiSe_2$ precursor is attributed to O 1s, associated with the oxidation in the air. It is noteworthy that NiO cannot convert to $Ni(OH)_2$ under the same preparation condition (**Fig. S4b**). The disappeared characteristic peaks of Se 3s/3p/3d/auger and enhanced characteristic peaks of O 1s in $NiSe_2/Ni(OH)_2$-2h indicate the formation of $Ni(OH)_2$.

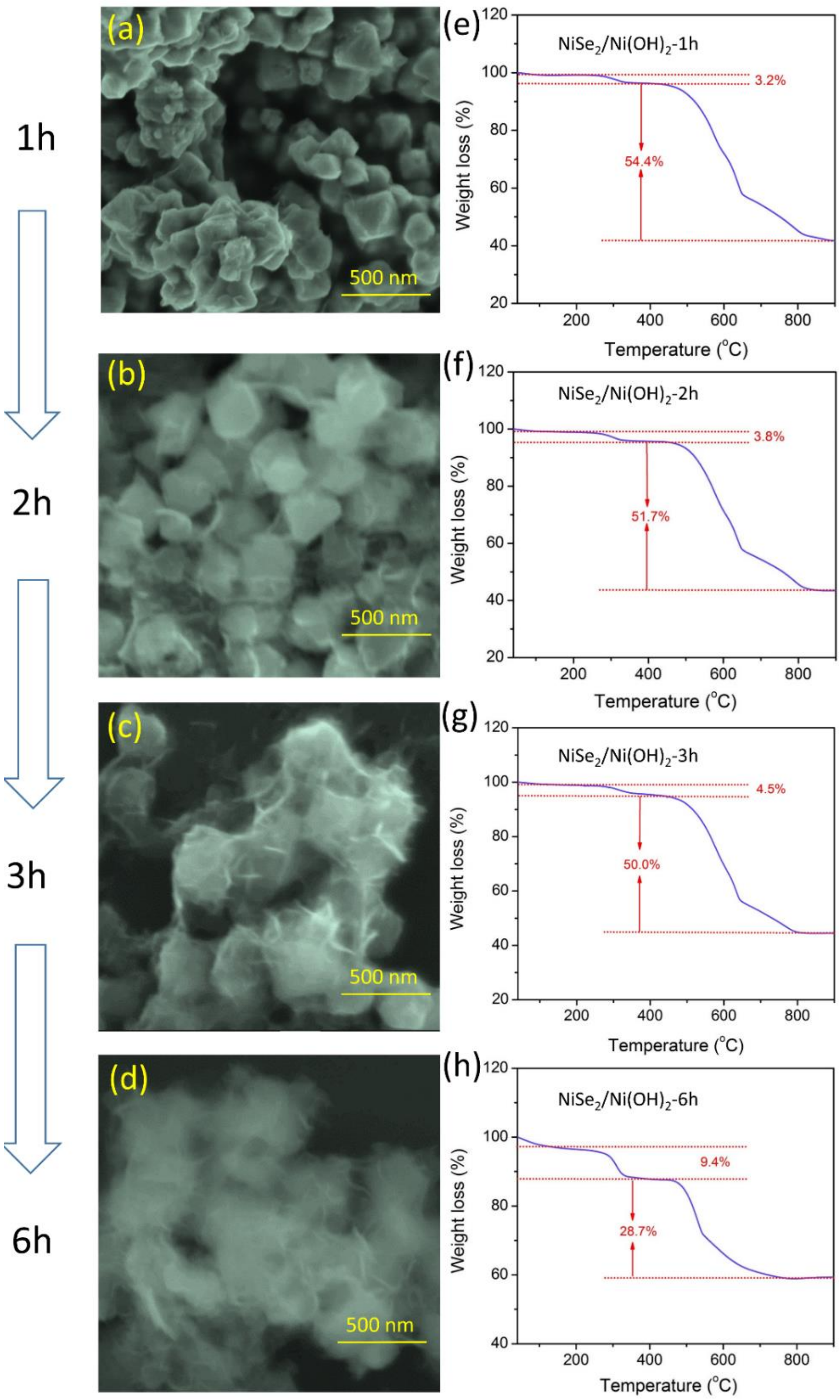

Fig. S6 (**a-d**) SEM images and (**e-f**) TGA curves of $NiSe_2/Ni(OH)_2$-1h, $NiSe_2/Ni(OH)_2$-2h, $NiSe_2/Ni(OH)_2$-3h, and $NiSe_2/Ni(OH)_2$-6h

In this work, TGA tests were employed to investigate the mass ratio of $NiSe_2$ and $Ni(OH)_2$ in $NiSe_2/Ni(OH)_2$ composites. The TGA measurements were under the O_2 condition in the temperature range from 40 to 900 °C. The corresponding reactions during hydrolysis are as follow:

$$Ni(OH)_2 \rightarrow NiO + H_2O \text{ (volatile)}$$

$$NiSe_2 + O_2 \rightarrow NiO + SeO_x \text{ (volatile)}$$

Herein, the molecular mass of $Ni(OH)_2$, $NiSe_2$ and NiO are 92.71, 216.61 and 74.71, respectively. The dehydration of $Ni(OH)_2$ and oxidation of $NiSe_2$ occurred in the temperature range from 40 to 400 °C and from 400 to 900 °C, respectively. Assuming the mass percentages of $Ni(OH)_2$ and $NiSe_2$ are M and N in a $NiSe_2/Ni(OH)_2$ composite, the TGA value changes can be calculated based on the following equations:

$$M \frac{216.61-74.71}{216.61}, \text{ from 40 to 400 °C}$$

$$N \frac{92.71-74.71}{92.71}, \text{ from 400 to 900 °C}$$

In $NiSe_2/Ni(OH)_2$-2h, the TGA value decreased 3.8% from 40 to 400 °C and 51.7% from 400 to 900 °C. Therefore, the calculated mass percentages of $NiSe_2$ and $Ni(OH)_2$ are 78.9% and 19.6% respectively. It is worth mentioned that the summation of the two values are a bit lower than 100%, and we suggest it is due to small amount of adsorbed water. Thus, the molar ratio between $NiSe_2$ and $Ni(OH)_2$ can be calculated:

$$NiSe_2 : Ni(OH)_2 = \frac{78.9\%}{216.61} : \frac{19.6\%}{92.71} = 1.72 : 1$$

The mass percentages of $NiSe_2$ and $Ni(OH)_2$ and molar ratios between them within all electrode materials are calculated, and the results are shown in the **Table S1**.

Table S1 The calculated mass percentages of $NiSe_2$ and $Ni(OH)_2$ and the molar ratios between them based on the TGA results

	**Mass percentage of $NiSe_2$**	**Mass percentage of $Ni(OH)_2$**	**Molar ratio between $NiSe_2$ and $Ni(OH)_2$**
**$NiSe_2/Ni(OH)_2$-1h**	83.1%	16.5%	2.16 : 1
**$NiSe_2/Ni(OH)_2$-2h**	78.9%	19.6%	1.72 : 1
**$NiSe_2/Ni(OH)_2$-3h**	76.3%	23.2%	1.41 : 1
**$NiSe_2/Ni(OH)_2$-6h**	43.8%	48.4%	0.387 : 1

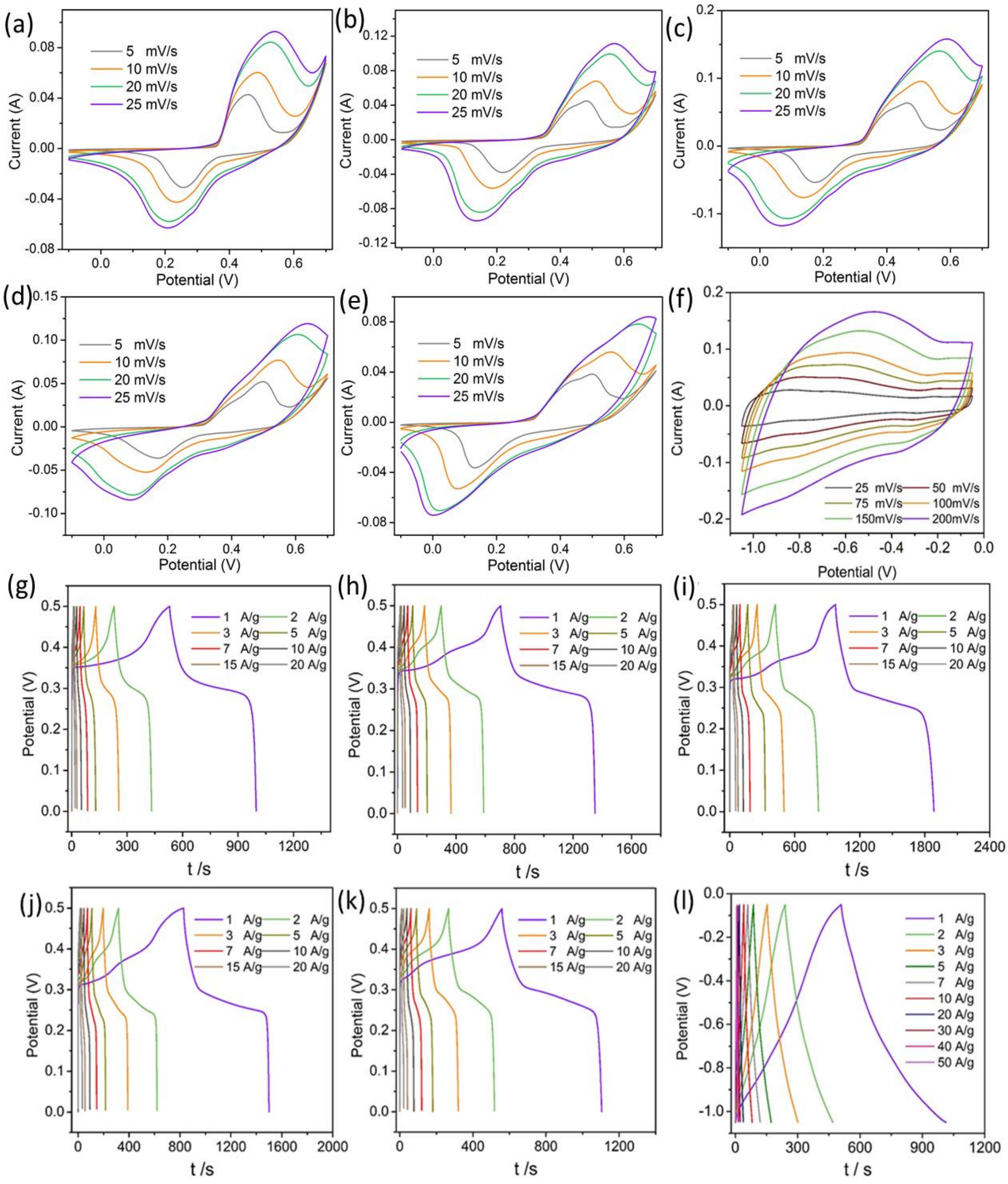

Fig. S7 (**a-f**) CV curves of $NiSe_2$, $NiSe_2/Ni(OH)_2$-1h, $NiSe_2/Ni(OH)_2$-2h, $NiSe_2/Ni(OH)_2$-3h, $NiSe_2/Ni(OH)_2$-6h, and PPD-rGO; (**g-l**) GCD curves of $NiSe_2$, $NiSe_2/Ni(OH)_2$-1h, $NiSe_2/Ni(OH)_2$-2h, $NiSe_2/Ni(OH)_2$-3h, $NiSe_2/Ni(OH)_2$-6h, and PPD-rGO

Table S2 Impedance fitting results of electrode materials

	Rs	CPE-T	CPE-P	Rct	Wo-R	Wo-T	Wo-P
**$NiSe_2$**	0.499	0.0267	0.818	0.521	1.594	0.919	0.433
**$NiSe_2/Ni(OH)_2$-1h**	0.473	0.0642	0.822	0.309	1.252	0.811	0.483
**$NiSe_2/Ni(OH)_2$-2h**	0.566	0.0934	0.859	0.222	0.601	0.703	0.475
**$NiSe_2/Ni(OH)_2$-3h**	0.526	0.0359	0.907	0.318	0.899	0.646	0.479
**$NiSe_2/Ni(OH)_2$-6h**	0.648	0.0329	0.899	0.304	0.958	0.524	0.467

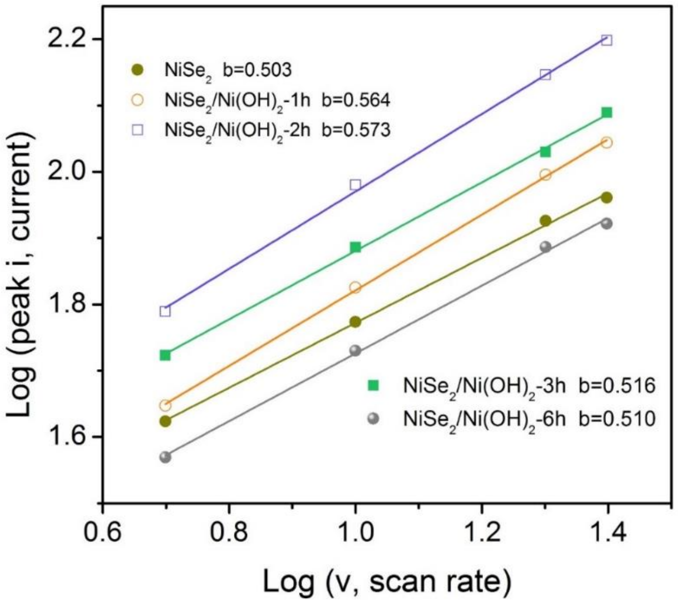

Fig. S8 b-value calculated from the oxide peak current. The fitting formula is: $i = a * v^b$. It is clearly that all electrode materials present distinct battery-type behaviors. The b-values for all electrodes are close to 0.5, indicating their battery-type behavior.

Table S3 Calculated b-value of prepared electrode

	a	b	Reduced chi-sqr
**$NiSe_2$**	0.604	0.503	$1.64E^{-8}$
**$NiSe_2/Ni(OH)_2$-1h**	0.891	0.564	$8.07E^{-7}$
**$NiSe_2/Ni(OH)_2$-2h**	1.315	0.573	$3.013E^{-6}$
**$NiSe_2/Ni(OH)_2$-3h**	0.817	0.516	$2.20E^{-6}$
**$NiSe_2/Ni(OH)_2$-6h**	0.541	0.510	$1.78E^{-6}$

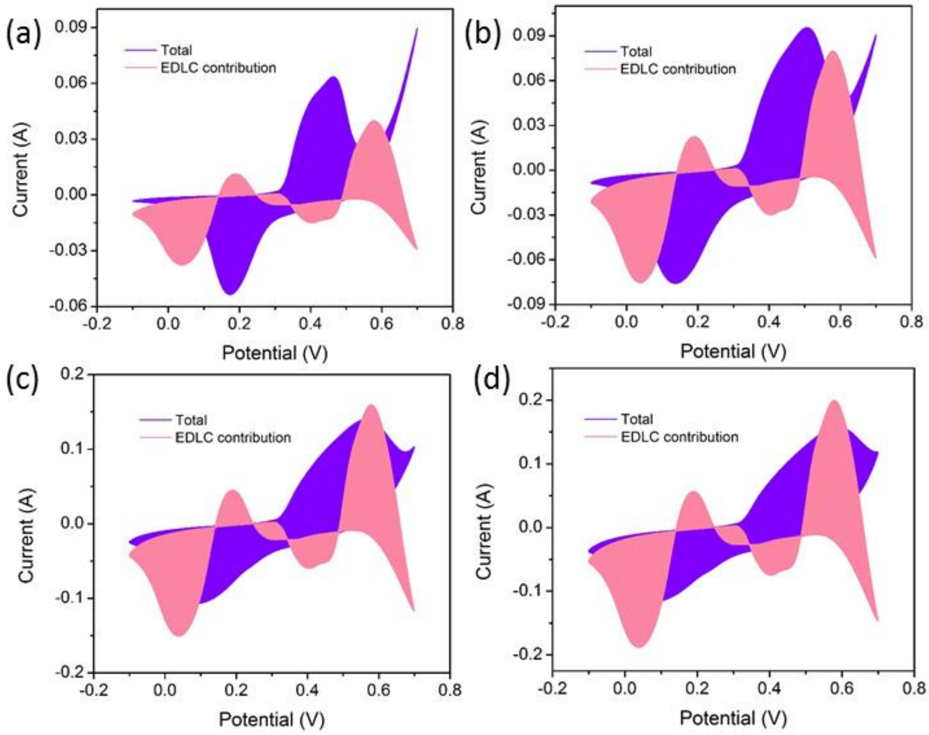

Fig. S9 (**a-d**) Calculated EDLC contribution of $NiSe_2/Ni(OH)_2$-2h by a traditional method under a scan rate of 5, 10, 20, and 25 mV s^{-1}. The strange shapes for all electrodes indicate that the equation of $i = k_1v + k_2v^{0.5}$ cannot be applied to the whole CV.

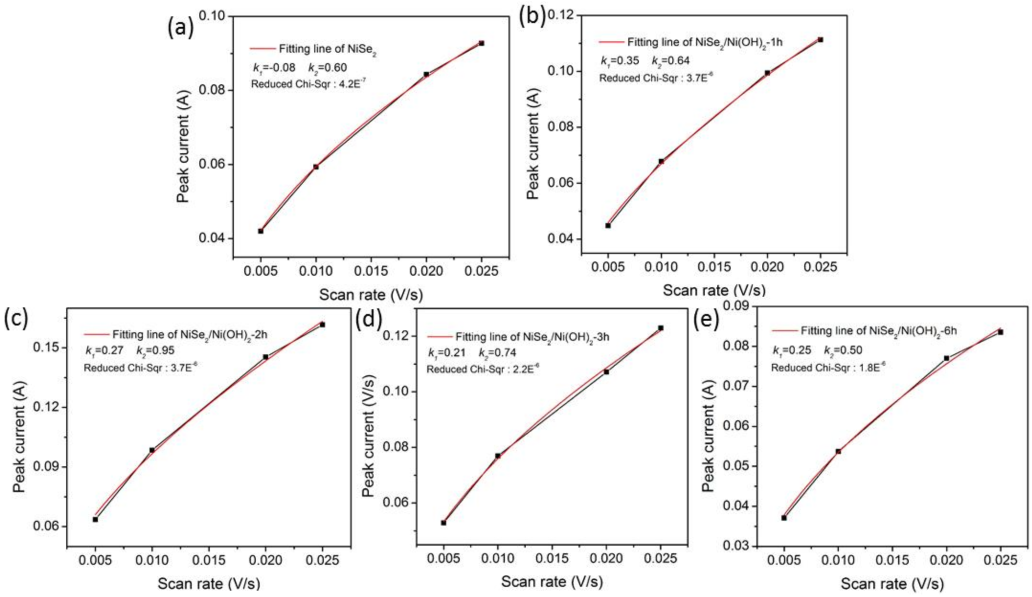

Fig. S10 (**a-e**) Fitting line of $NiSe_2$, $NiSe_2/Ni(OH)_2$-1h, $NiSe_2/Ni(OH)_2$-2h, $NiSe_2/Ni(OH)_2$-3h and $NiSe_2/Ni(OH)_2$-6h; The formula of the line is : $i = k_1v + k_2v^{0.5}$. The small reduced Chi-suqr values indicate the reasonable fitting results for all electrodes.

Table S4 Performance comparison of electrode materials

Materials	Specific capacity	Cycling performance	journal
**$NiSe_2/Ni(OH)_2$**	**909 C/g at 1 A/g (1818 F/g)**	**85% retention after 5000 cycles**	**This work**
Ni/Co-LDH	826 C/g at 1 A/g (1652 F/g)	100% retention after 3000 cycles	[S1]
$NiSe_2$	417.6 C/g at 3 A/g (1044 F/g)	67% retention after 2000 cycles	[S2]
Ag-rGO/$Ni(OH)_2$	520 C/g at 2 A/g (1040 F/g)	92.6% retention after 2000 cycles	[S3]
$(Ni,Co)Se_2$/NiCo-LDH	612 C/g at 2 A/g (1224 F/g)	89% retention after 3000 cycles	[S4]
$NiCo_2S_4$-C	468 C/g at 1 A/g (932 F/g)	94% retention after 3000 cycles	[S5]
$NiCoSe_2$	450 C/g at 3 A/g (750 F/g)	89% retention after 5000 cycles	[S6]
$Ni(OH)_2$	713.2 C/g at 1 A/g	65% retention after 4500 cycles	[S7]

Table S5 Capacity contribution of $NiSe_2$ and $Ni(OH)_2$

	**Mass percentage of $NiSe_2$**	**SC (5 A/g)**	**Capacity contribution of $NiSe_2$**	**Capacity contribution of $Ni(OH)_2$**
**$NiSe_2$**	1	323	323	0
**$NiSe_2/Ni(OH)_2$-1h**	83.10%	497	268	229
**$NiSe_2/Ni(OH)_2$-2h**	78.90%	690	254	436
**$NiSe_2/Ni(OH)_2$-3h**	76.30%	557	246	311
**$NiSe_2/Ni(OH)_2$-6h**	43.80%	442	141	301

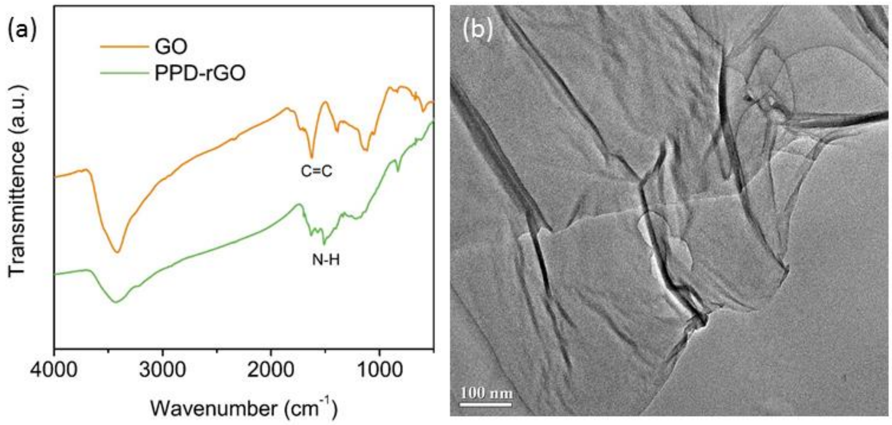

Fig. S11 (**a**) FT-IR spectrum of GO and PPD-rGO; (**b**) TEM image of PPD-rGO

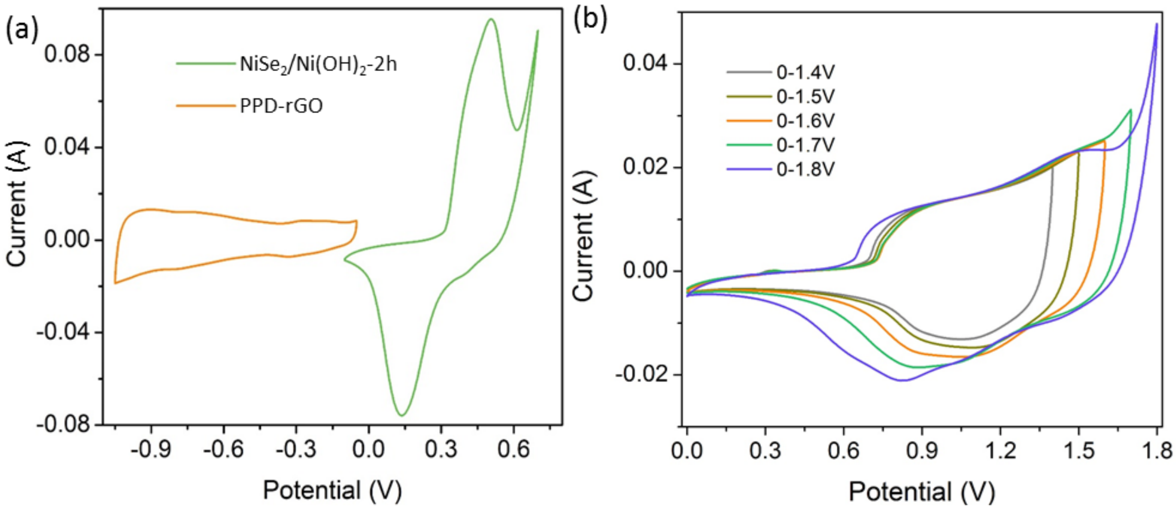

Fig. S12 (**a**) CV curves of PPD-rGO and $NiSe_2/Ni(OH)_2$-2h at 10 mV/s; (**b**) CV curves of assembled button asymmetric supercapacitor at different voltages

Table S6 Performance comparison of asymmetric supercapacitor

	Energy density	Cycling performance	journal
**Ni-Se-OH//PPD-rGO**	**76.1 Wh/Kg at 906 W/Kg**	**82% retention after 8000 cycles**	**This work**
NiCoP/NiCo-OH//AC	34 Wh/Kg at 775 W/Kg	92% retention after 1000 cycles	[S8]
$NiSe_2$//AC	44.8 Wh/Kg at 969.7 W/Kg	87.4% retention after 20000 cycles	[S2]
$NiCo_2O_4$//AC	69.7 Wh/Kg at 373.9 W/Kg	90.3% retention after 5000 cycles	[S9]
$Ni(OH)_2$//AC	34 Wh/Kg at 756 W/Kg	91.5% retention after 6000 cycles	[S10]
Cu_3SbS_4/Ni-$_5$//Cu_2MoS4/Ni	58.1 Wh/Kg at 636.36 W/Kg	90.7% retention after 4000 cycles	[S11]
ZnNiCo-P//PPD-rGOs	60.1 Wh/Kg at 960 W/Kg	89% retention after 8000 cycles	[S12]
CoNi-MOF//AC	28.5 Wh/Kg at 1500 W/Kg	94% retention after 5000 cycles	[S13]
$NiCoS_2$//AC	38.64 Wh/Kg at 1330 W/Kg	99.3% retention after 5000 cycles	[S14]
Ag-rGO/$Ni(OH)_2$//AC	41.2 Wh/Kg at 375 W/Kg	90.4% retention after 2000 cycles	[S3]

Supplementary References

[S1] Z. Xiao, Y. Mei, S. Yuan, H. Mei, B. Xu et al., Controlled hydrolysis of metal–organic frameworks: hierarchical Ni/Co-layered double hydroxide microspheres for high-performance supercapacitors. ACS Nano **13**(6), 7024-7030 (2019). https://doi.org/10.1021/acsnano.9b02106

[S2] S. Wang, W. Li, L. Xin, M. Wu, Y. Long, H. Huang, X. Lou, Facile synthesis of truncated cube-like $NiSe_2$ single crystals for high-performance asymmetric supercapacitors. Chem. Eng. J. **330**, 1334-1341 (2017). https://doi.org/10.1016/j.cej.2017.08.078

[S3] E. Cho, C. Chang-Jian, K. Lee, J. Huang, B. Ho, R. Liu, Y. Hsiao, Ternary composite based on homogeneous $Ni(OH)_2$ on graphene with ag nanoparticles as nanospacers for efficient supercapacitor. Chem. Eng. J. **334**, 2058-2067 (2018). https://doi.org/10.1016/j.cej.2017.11.175

[S4] X. Li, H. Wu, C. Guan, A.M. Elshahawy, Y. Dong, S.J. Pennycook, J. Wang, (Ni, Co)Se_2/NiCo-LDH core/shell structural electrode with the cactus-like (Ni,Co)Se_2 core for asymmetric supercapacitors. Small 1803895 (2018). https://doi.org/10.1002/smll.201803895

[S5] Y. Zhang, Y. Zhang, Y. Zhang, H. Si, L. Sun, Bimetallic $NiCo_2S_4$ nanoneedles anchored on mesocarbon microbeads as advanced electrodes for asymmetric supercapacitors. Nano-Micro Letters. **11**, 1 (2019). https://doi.org/10.1007/s40820-019-0265-1

[S6] L. Hou, Y. Shi, C. Wu, Y. Zhang, Y. Ma et al., Monodisperse metallic $NiCoSe_2$ hollow sub-microspheres: formation process, intrinsic charge-storage mechanism, and appealing pseudocapacitance as highly conductive electrode for electrochemical supercapacitors. Adv. Funct. Mater. **28**(13), 1705921 (2018). https://doi.org/10.1002/adfm.201705921

[S7] S. Zhang, Z. Yang, K. Gong, B. Xu, H. Mei, H. Zhang, J. Zhang, Z. Kang, Y. Yan, D. Sun, Temperature controlled diffusion of hydroxide ions in 1d channels of Ni-MOF-74 for its complete conformal hydrolysis to hierarchical $Ni(OH)_2$ supercapacitor electrodes. Nanoscale **11**(19), 9598-9607 (2019). https://doi.org/10.1039/C9NR02555C

[S8] X. Li, H. Wu, A.M. Elshahawy, L. Wang, S.J. Pennycook, C. Guan, J. Wang, Cactus-like NiCoP/NiCo-OH 3d architecture with tunable composition for high-performance electrochemical capacitors. Adv. Funct. Mater. **28,** 1800036 (2018) 1800036. https://doi.org/10.1002/adfm.201800036

[S9] Y. Xue, T. Chen, S. Song, P. Kim, J. Bae, Dna-directed fabrication of $NiCo_2o_4$ nanoparticles on carbon nanotubes as electrodes for high-performance battery-like electrochemical capacitive energy storage device. Nano Energy **56**, 751-758 (2019). https://doi.org/10.1016/j.nanoen.2018.11.003

[S10] W. He, G. Zhao, P. Sun, P. Hou, L. Zhu, T. Wang, L. Li, X. Xu, T. Zhai, Construction of longan–like hybrid structures by anchoring nickel hydroxide on yolk–shell polypyrrole for asymmetric supercapacitors. Nano Energy **56,** 207-215 (2019). https://doi.org/10.1016/j.nanoen.2018.11.048

[S11] V.K. Mariappan, K. Krishnamoorthy, P. Pazhamalai, S. Sahoo, S.S. Nardekar, S. Kim, Nanostructured ternary metal chalcogenide-based binder-free electrodes for high energy density asymmetric supercapacitors. Nano Energy **57,** 307-316 (2019). https://doi.org/10.1016/j.nanoen.2018.12.031

[S12] J. Li, Z. Liu, Q. Zhang, Y. Cheng, B. Zhao, S. Dai, H. Wu, K. Zhang, D. Ding, Y. Wu, M. Liu, M. Wang, Anion and cation substitution in transition-metal oxides nanosheets for high-performance hybrid supercapacitors. Nano Energy **57**, 22-33 (2019). https://doi.org/10.1016/j.nanoen.2018.12.011

[S13] T. Deng, Y. Lu, W. Zhang, M. Sui, X. Shi, D. Wang, W. Zheng, Inverted design for high-performance supercapacitor via $Co(OH)_2$-derived highly oriented MOF electrodes. Adv. Energy Mater. **8**(7), 1702294 (2018). https://doi.org/10.1002/aenm.201702294

[S14] W. He, C. Wang, H. Li, X. Deng, X. Xu, T. Zhai, Ultrathin and porous $Ni_3S_2/CoNi_2S_4$ 3d-network structure for super high energy density asymmetric supercapacitors. Adv. Energy Mater. **7**(21), 1700983 (2017). https://doi.org/10.1002/aenm.201700983
